# Erratum to “Identification, characterization, and expression of *Oryza sativa* tryptophan decarboxylase genes associated with fluroxypyr‐meptyl metabolism”

**DOI:** 10.1002/tpg2.70026

**Published:** 2025-03-28

Wang, H. W., Shi, X. Z., Zhong, X. Y., Ai, G., Wang, Y. H., Zhou, Z. Z., Lu, D., Liu, X. L., & Chen, Z. J. (2025). Identification, characterization, and expression of *Oryza sativa* tryptophan decarboxylase genes associated with fluroxypyr‐meptyl metabolism. *The Plant Genome, 18*, e20547. https://doi.org/10.1002/tpg2.20547

A corresponding author was not included in the correspondence information. The correct correspondence information is “Xiao Liang Liu and Zhao Jie Chen, Guangxi Key Laboratory of Agric‐Environment and Agric‐Products Safety, College of Agriculture, Guangxi University, Nanning, Guangxi, 530004, China”. Email: liuxiaoliang2007@163.com, zhaojie-chen@gxu.edu.cn

We apologize for this.

